# Bibliometric and visualization analysis of literature relating to diabetic erectile dysfunction

**DOI:** 10.3389/fendo.2022.1091999

**Published:** 2022-12-09

**Authors:** Fanchao Meng, Xiaoxing Liao, Haimin Chen, Sheng Deng, Lu Wang, Mengjie Zhao, Haibin Li, Dong Liu, Guojing Gao, Haisong Li, Jisheng Wang

**Affiliations:** ^1^ Urology Surgery, The Third Affiliated Hospital of Beijing University of Chinese Medicine, Beijing, China; ^2^ Department of Nephroendocrinology, Dongzhimen Hospital, Beijing University of Chinese Medicine, Beijing, China; ^3^ Department of Andrology, Shunyi Hospital, Beijing Hospital of Traditional Chinese Medicine, Beijing, China; ^4^ Department of Surgery, Beijing Xuanwu Traditional Chinese Medicine Hospital, Beijing, China; ^5^ Department of Andrology, Dongzhimen Hospital, Beijing University of Chinese Medicine, Beijing, China

**Keywords:** diabetic erectile dysfunction, bibliometric analysis, CiteSpace, VOSviewer, visualization

## Abstract

**Introduction:**

Diabetic erectile dysfunction (DMED) refers to erectile dysfunction secondary to diabetes. Erectile dysfunction is characterized by a persistent inability to achieve and maintain an erection sufficient to permit satisfactory sexual activity.

**Methods:**

Based on the Web of Science core collection database, we firstly analyzed the quantity and quality of publications in the field of DMED, secondly profiled the publishing groups in terms of country, institution, author’s publication and cooperation network, and finally sorted out and summarized the hot topics of research.

**Results:**

From 2001 to 2022, a total of 1,403 articles relating to this topic were published in 359 journals. They represent the global research status, potential hotspots, and future research directions. The number of DMED-related publications and citations has steadily increased over the few past decades. Academic institutions from Europe and the United States have played a leading role in DMED research. The country, institution, journal, and author with the most publications were the United States (294), INHA University (39), the Journal of Sexual Medicine (156), and Ryu, Ji-Kan (29), respectively. The most common keywords were erectile dysfunction (796), men (256), diabetes (254), diabetes mellitus (239), prevalence (180), corpus cavernosum (171), dysfunction (155), mellitus (154), nitric-oxide synthase (153), and expression (140). The main keyword-based research topics and hotspots in the DMED field were oral sildenafil, smooth muscle relaxation, nitric oxide synthase, gene therapy, metabolic syndrome, cavernous nerve injury, stem cell, and penile prosthesis.

**Discussion:**

The terms oral sildenafil, smooth muscle relaxation, nitric oxide synthase, gene therapy, metabolic syndrome, cavernous nerve injury, stem cell, and penile prosthesis will be at the forefront of DMED-related research.

## Introduction

Diabetes is a chronic, non-infectious disease caused by both genetic and environmental factors. Over the past three decades, the number of people with diabetes worldwide has more than doubled, making it one of the most important global public health challenges (1). Erectile dysfunction is defined as the persistent inability to achieve and maintain sufficient erectile capacity to permit satisfactory sexual behavior (2). Diabetic erectile dysfunction (DMED) refers to erectile dysfunction secondary to diabetes mellitus (3). The incidence of erectile dysfunction ranges from 0.1% to 18% in the normal population but is nearly three-fold higher in patients with diabetes, and affected people tend to be younger (4).

There is evidence for a link between diabetes and the development of erectile dysfunction in both animal models and humans (5). The central nervous system and pericardial nerve damage caused by a high-glucose environment is an important cause of DMED (6). In addition, age, duration of diabetes, blood glucose control, smoking, hypertension, atherosclerosis, adverse drug reactions, and psychological factors are all closely related to the occurrence of DMED. Current treatment methods for DMED can be summarized as primary disease treatment, psychological treatment, and symptomatic treatment.

The term bibliometrics was coined by Alan Pritchard in 1969 (7). Bibliometric analysis is a powerful tool that uses literature measures or indicators to quantify research performance in a given field (8, 9). CiteSpace and VOSviewer are commonly used processing tools for visualizing research impact based on co-word, co-citation, and literature-coupling analysis (10).

Based on the advantages of clustering technology and map presentation, the research trend of any given field can be analyzed and displayed as a multivariate, comprehensive, visual knowledge map (11, 12). Using such bibliometric software, literature related to DMED in recent decades can be visually displayed and analyzed. Accordingly, with Scopus as the data source, we utilized CiteSpace, VOSviewer, and Microsoft Excel to show the knowledge base, development trends, and emerging hotspots in the DMED field.

## Materials and methods

### Ethics statement

No approval was required from the Institutional Review Board as data were retrieved from the Web of Science (WOS) database (https://www.webofscience.com/wos/woscc/basic-search) and no human subjects were involved.

### Sources and collection

The WOS is the most commonly used database in scientific and bibliometric research. It contains nearly 9,000 of the world’s most prestigious high-impact journals and more than 12,000 academic conferences, thus providing a comprehensive overview of the global research results in science, technology, medicine, and other fields (13, 14).

Publication information in the DMED area was searched within one day to ensure that no data were updated. The period was January 1, 2001, through September 30, 2022, and the retrieval date was October 1, 2022. The WOS Core Collection was selected, the subject words were “Diabetic erectile dysfunction”, and the article types were “article” and “review”. The retrieved results were exported in “Plain text file” format, and “Full Record and Cited References” was selected for “Record content”.

The search query string was described as follows: Results for “diabetic erectile dysfunction” (Topic) and Article or Review Article (Document Type) and Book Chapters (Exclude - Document Type).

### Bibliometric analysis and software

CiteSpace software (Drexel University, Philadelphia, PA, USA), designed by Dr. Chen Chaomei in 2004 (11), is a freely available Java application widely used for the visualization and analysis of trends and patterns in scientific literature (15). CiteSpace (https://citespace.podia.com/download, R6.1.3) to scientometrics, data and information visualization technology as the foundation, through the analysis of the potential knowledge of literature, regularity and distribution, present knowledge structure. In this study, CiteSpace was used for keyword clustering and salient word analysis.

VOSviewer 1.6.18 (https://www.vosviewer.com/) is a software tool for constructing and visualizing bibliometric networks (16). In this study, VOSviewer was used to visualize countries/regions, authors, institutional collaborations, citations, and keyword co-occurrence, as well as to construct density maps.

The aim was to describe all the literature characteristics, including country/institution, journals, highly cited articles, cluster network of co-cited references, and most frequently cited keywords. In particular, in addition to noun phrases extracted from article titles and abstracts, burst detection was applied to the keywords of publications in the article collection assigned to the citation extension.

## Results

### Time trends in publications and citations

**Figure 1:**
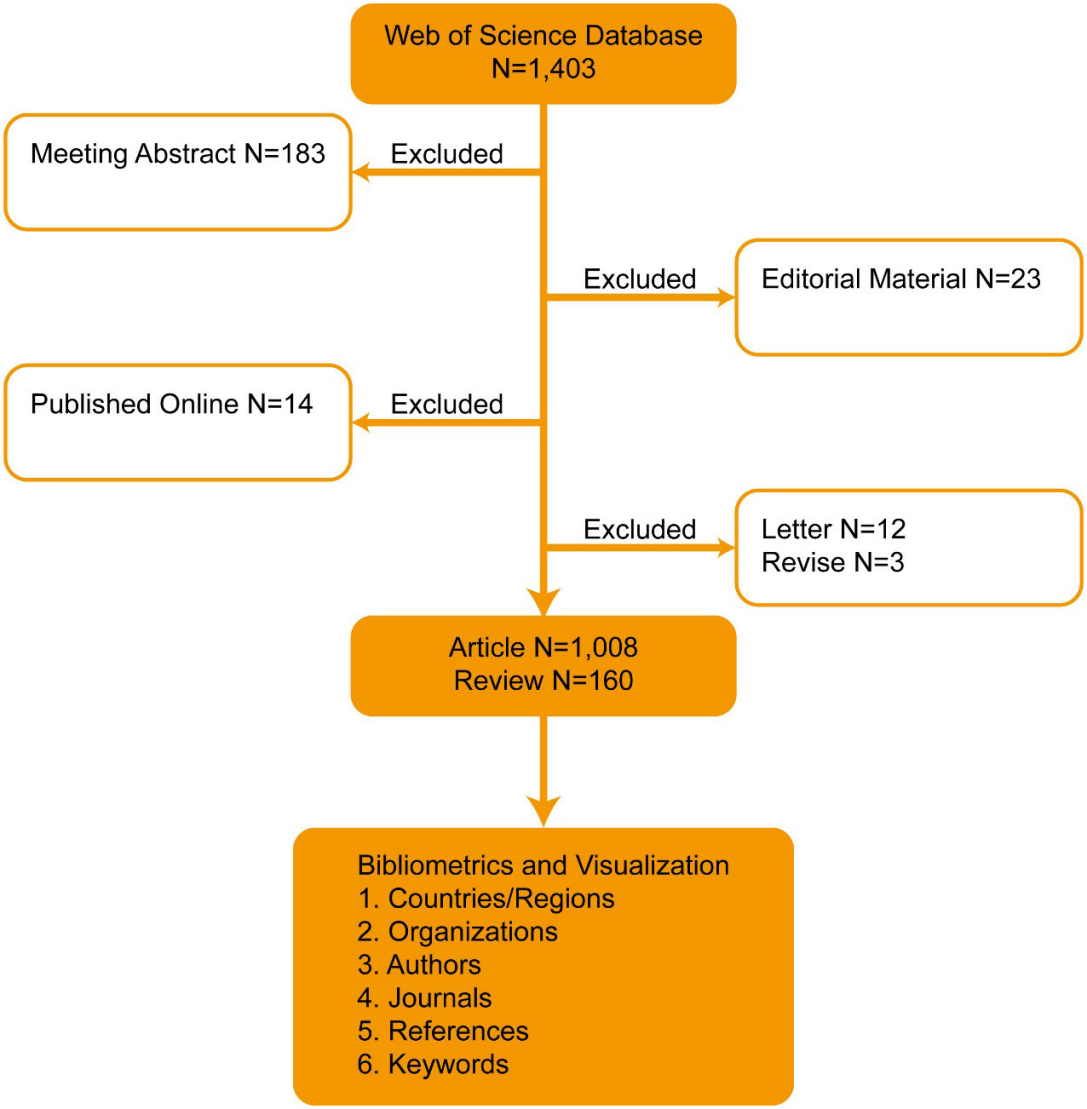
Search flowchart.

**Figure 2:**
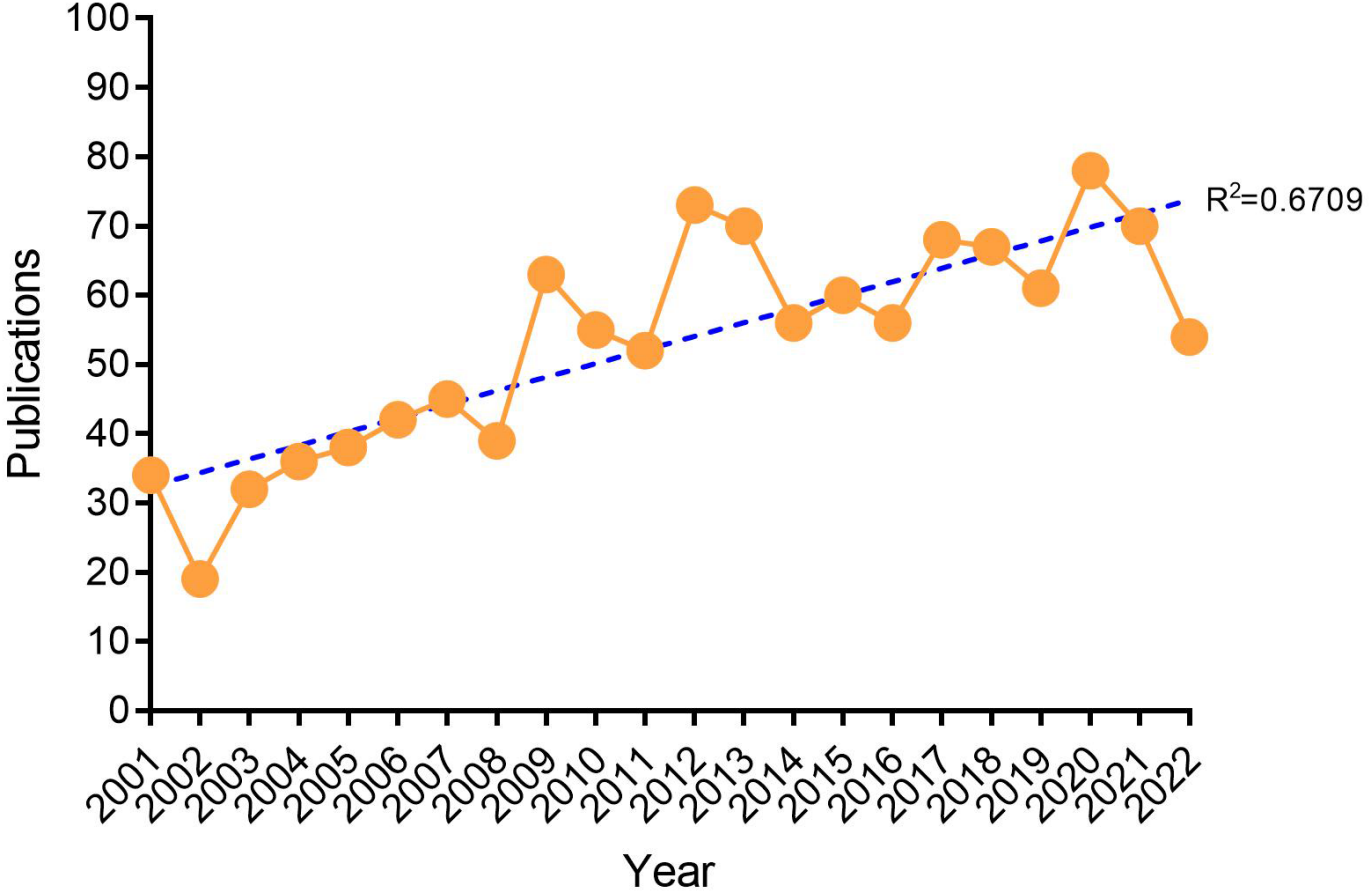
Annual trends of global publications.

The number of annual publications is an important parameter for evaluating the development of scientific research and can reflect the growth of knowledge in this field to a certain extent. As of September 30, 2022, a total of 1,403 publications related to DMED were published (**Figure 1**). The number of articles published per year is shown in **Figure 2**. Although the trend was upward overall, it fluctuated slightly in some years. Notably, the number of publications per year can be easily divided into two phases. In the first phase, from 2001 to 2008, the average number of publications per year was around 30; in the second phase, from 2009 to 2022, the average number of publications per year stabilized at more than 50. Overall, the amount of knowledge in the DMED research field showed a linear growth trend (*R* ^2 =^ 0.6709), reflecting the increasing research interest in this field.

### Analysis of the most productive countries/regions

**Figure 3:**
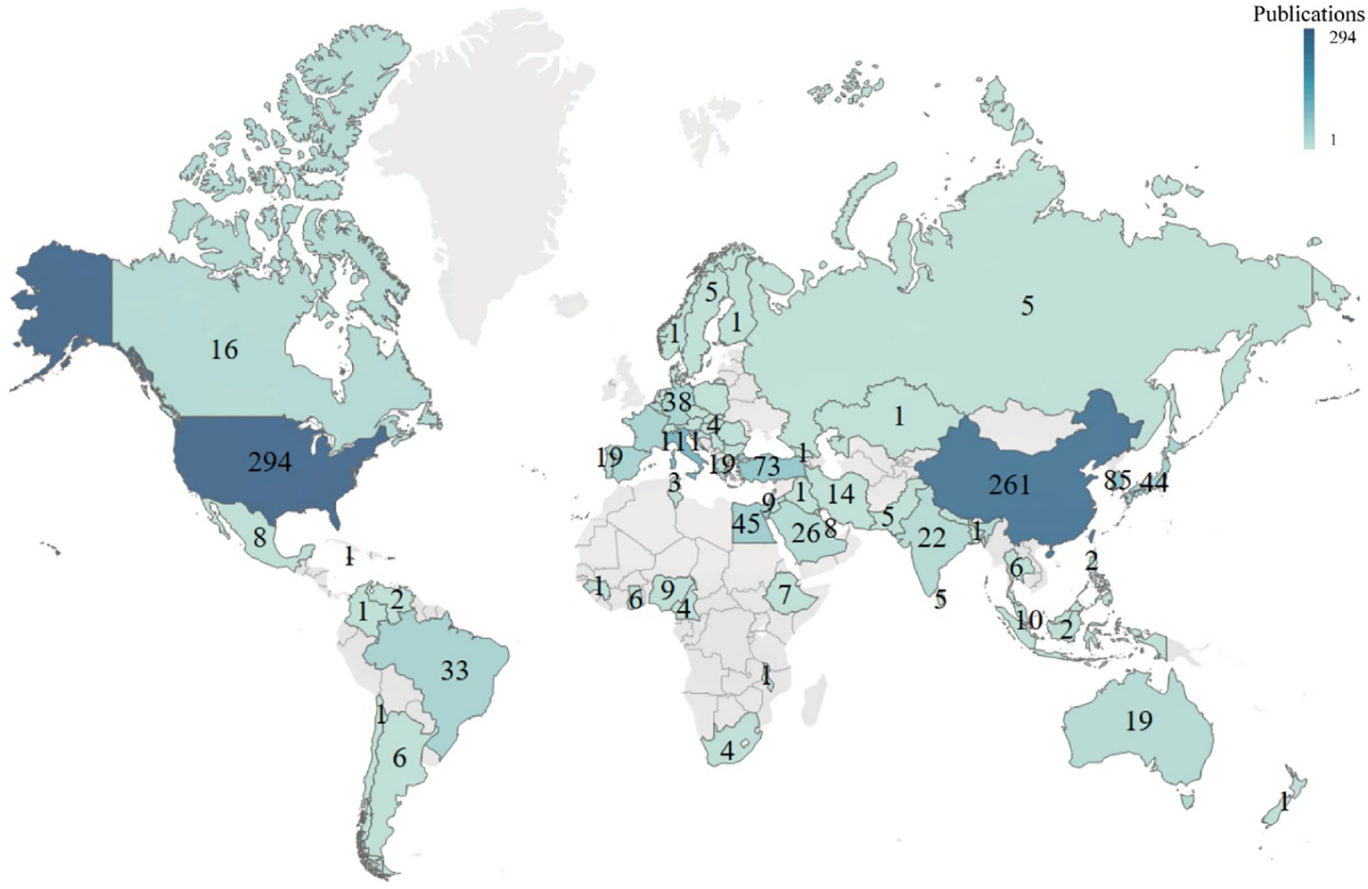
Analysis of the global research trends based on the origins of the publications.

A total of 75 countries/regions have published articles in this field. The 10 countries that made the most significant contributions to DMED-related publications were the USA (294), China (244), Italy (111), South Korea (85), England (79), Turkey (73), Egypt (45), Japan (44), Spain (40), and Germany (38) (**Figure 3**; **Table 1**). The size of the nodes is determined by the number of publications (the larger the number, the larger the node). The same colors represent the same clusters. The lines between nodes represent the alignment between countries/regions (the stronger the partnership, the wider the boundaries). The total link strength reflects the combined strength between countries/regions. As shown in **Table 2**, the USA had the largest number of publications, the highest number of citations (15,620), and the greatest link strength (164). The USA (15,620), Italy (6,917), England (6,645), China (4,495), and Germany (2,381) were the top five countries in terms of citations. The above results indicated that these countries exerted the greatest influence on DMED-related research.

**Table 1:** The 10 countries that contributed the most to DMED-related publications.

Rank	Country	Documents	Citations	Total link strength	H-index
1	USA	294	15620	164	51
2	China	244	4495	78	35
3	Italy	111	6917	66	40
4	South Korea	85	1510	32	24
5	England	79	6645	82	30
6	Turkey	73	844	31	15
7	Egypt	45	777	21	15
8	Japan	44	894	13	16
9	Spain	40	1280	35	17
10	Germany	38	2381	38	11

**Table 2:** The number of citations of publications on DMED in the top 10 countries.

Rank	Country	Documents	Citations	Total link strength
1	USA	294	15620	3542
2	Italy	111	6917	1150
3	England	79	6645	1219
4	China	244	4495	2638
5	Germany	38	2381	241
6	France	34	2358	436
7	Australia	19	2057	192
8	Canada	16	2053	322
9	South Korea	85	1510	1048
10	Spain	40	1280	532

**Figure 4:**
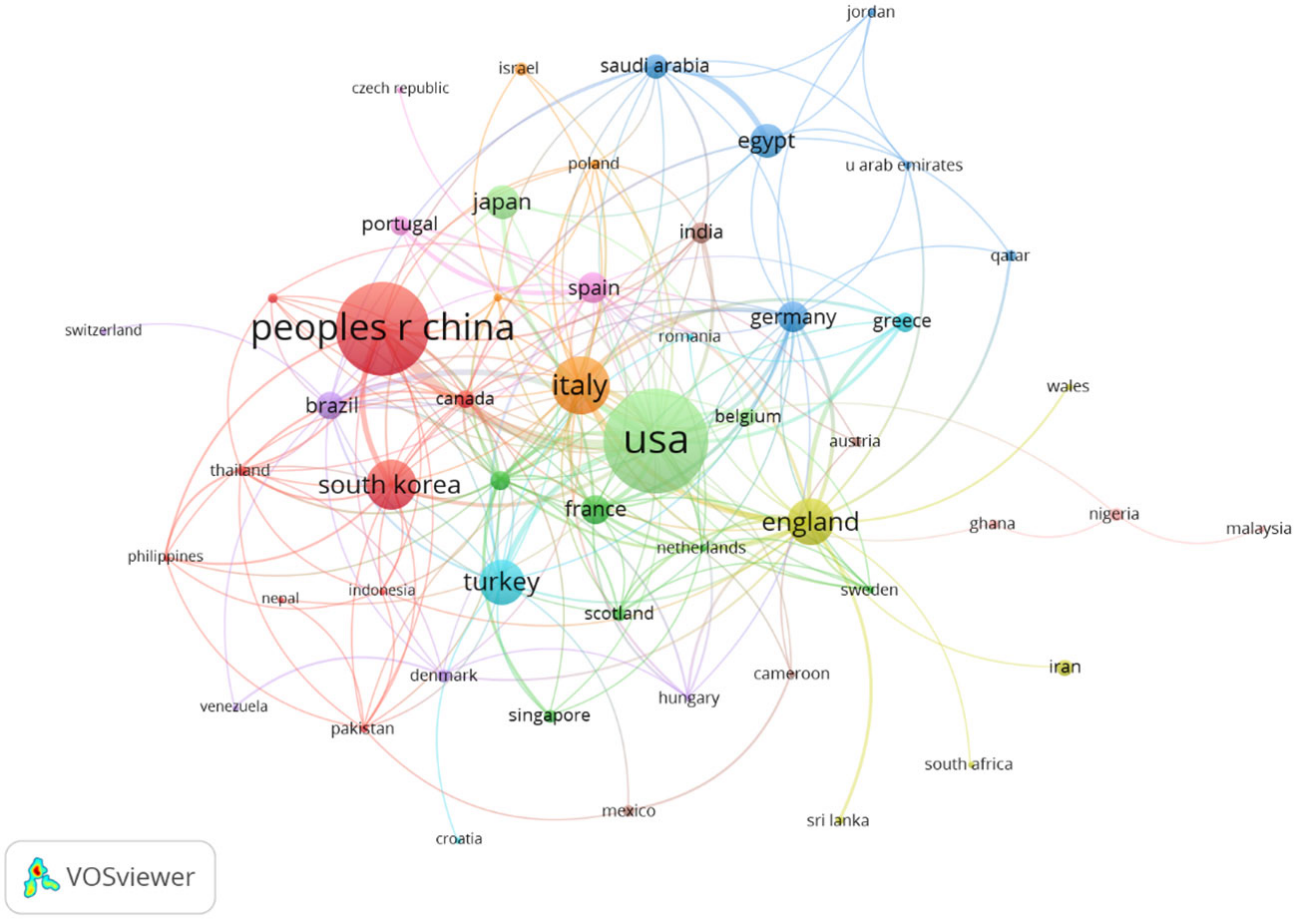
Co-occurrence map of countries/regions. The size of the nodes represents the number of articles. The thickness of the curves represents the strength of collaboration. The colors represent different collaboration groups.

VOSviewer was used to analyze cooperation across countries, with lines between nodes indicating co-authorship between countries (the thicker the line the stronger the cooperation). China, the USA, Italy, South Korea, and England cooperated the most with other countries (**Figure 4**).

### Contributions of the top organizations

**Figure 5:**
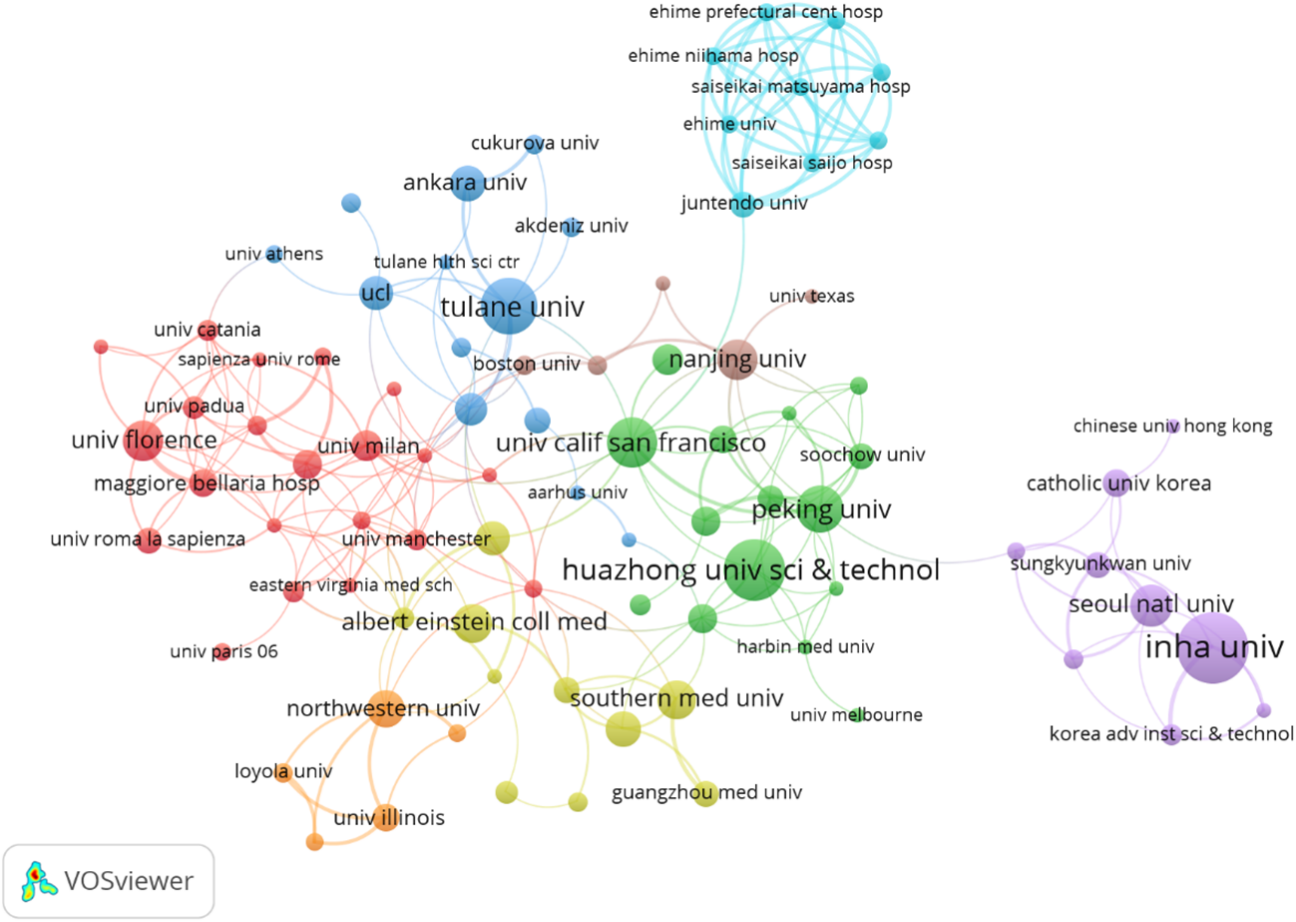
Co-occurrence map of institutions. The node size represents the number of articles; the curve thickness represents the strength of collaboration; the different collaboration groups are sorted by color.

A total of 1,465 institutions participated in the publication of papers related to DMED technology. The 10 institutions that contributed the most to DMED publications were INHA University (39), Huazhong University of Science & Technology (32), Tulane University (29), University of California San Francisco (25), Peking University (23), Seoul National University (20), Cairo University (20), University of Florence (19), Nanjing University (19), and Southern Medical University (18) (**Table 3**). These results demonstrated that INHA University had the greatest number of publications with the highest number of citations (663) and the greatest link strength (20). Each node represents a different institution. The size of the node is determined by the number of publications (the larger the number, the larger the node). The same colors represent the same clusters. Boundaries between nodes represent a collaboration between organizations (the stronger the partnership, the wider the boundaries). The total link strength reflects the aggregate strength between institutions. As can be seen from the map, 146 institutions actively cooperated both within and between clusters. The three institutions with the highest total link strength were Peking University (34), the University of Florence (24), and the University of California San Francisco (23). The five institutions with the greatest number of citations were Eastern Virginia Medical School (4,067), Harvard University (3,845), The University of Pavia (2,092), the University of Manchester (1,684), and the University of Rome Tor Vergata (1,558) (**Table 4**). Tulane University, University of Florence, University of California San Francisco, INHA University, and Huazhong University of Science & Technology were at the center of such collaborations. However, most institutions were fragmented and displayed little cooperation (**Figure 5**).

**Table 3:** The 10 institutions that contributed the most publications on assisted reproduction.

Rank	Organization	Documents	Citations	Total link strength
1	INHA University	39	663	20
2	Huazhong University of Science & Technology	32	414	6
3	Tulane University	29	1048	20
4	University of California San Francisco	25	1041	23
5	Peking University	23	618	34
6	Seoul National University	20	357	19
7	Cairo University	20	346	9
8	University of Florence	19	1532	24
9	Nanjing University	19	695	15
10	Southern Medical University	18	305	14

**Table 4:** The number of citations for publications on DMED by the top 10 institutions.

Rank	Organization	Documents	Citations	Total link strength
1	Eastern Virginia Medical School	5	4067	59
2	Harvard University	6	3845	63
3	University of Pavia	12	2092	207
4	University of Manchester	8	1684	100
5	University of Rome Tor Vergata	5	1558	55
6	University of Michigan	8	1543	67
7	University of Florence	19	1532	307
8	Mayo Clinic	5	1486	33
9	University of Dusseldorf	8	1403	58
10	John Hopkins University Hospital	14	1145	375

### Analysis of authors and co-cited authors

**Figure 6:**
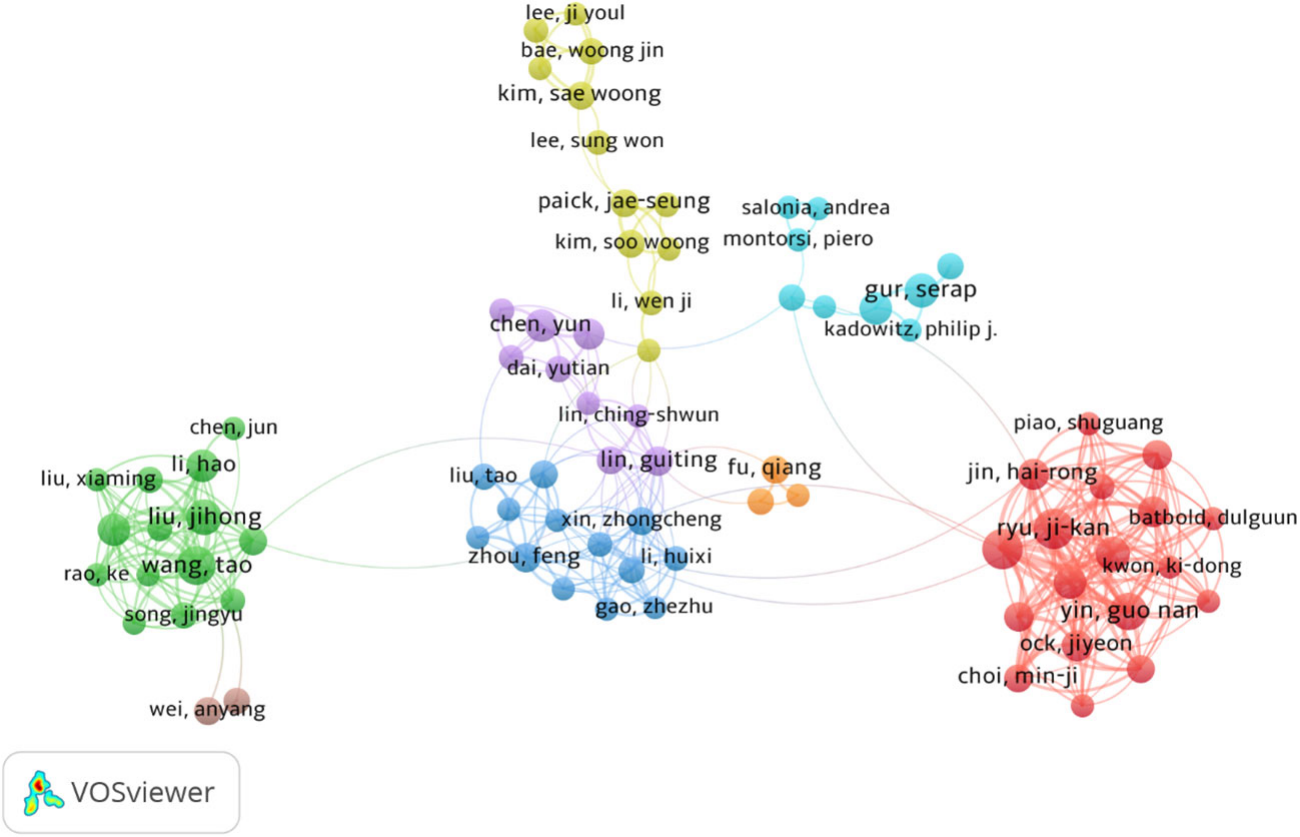
CiteSpace visualization map of authors involved in publications related to assisted reproduction. Nodes represent authors (the larger the circle, the greater the number of publications). The lines between nodes represent the cooperation between two authors of the same article (the wider the line, the more frequent the cooperation). The node color represents the year.

Author co-occurrence analysis can identify the core authors in a field and the strength of collaboration among authors. Co-citation means that two authors or papers are cited by a third author or paper at the same time. This study included 5,216 authors and 20,297 co-cited authors. Of these, Ji-Kan Ryu (29), Jun-Kyu Suh (29), Wang Tao (26), Yin Guo Nan (25), and Liu Jihong (20) published the most articles (**Table 5**). At the same time, Jihong Liu and Guiting Lin obtained the most cooperation, forming two stable author cooperation groups (**Figure 6**). However, there was a lack of cooperation among other authors and teams, and the research was in a relatively scattered state. The results of the co-citation relationship showed that Bivalacqua, TJ (430); Corona, G (340); Feldman, HA (259); Rosen, RC (258); and Burnett, AL (213) were the most frequently cited authors (**Table 6**), indicating that they play an important role in DMED research.

**Table 5:** The number of articles published by the top 10 authors.

Rank	Author	Documents	Citations	Total link strength
1	Ryu, Ji-Kan	29	483	189
2	Suh, Jun-Kyu	29	483	189
3	Wang, Tao	26	294	100
4	Yin, Guo Nan	25	334	174
5	Liu, Jihong	20	213	90
6	Song, Kang-Moon	17	274	142
7	Gur, Serap	17	154	19
8	Podlasek, Carol A	15	286	46
9	Hellstrom, Wayne J	15	309	13
10	Kwon, Mi-Hye	14	257	119

**Table 6:** The number of co-citations of the top 10 authors.

Rank	Author	Citations	Total link strength
1	Bivalacqua, TJ	430	6879
2	Corona, G	340	3315
3	Feldman, HA	259	2611
4	Rosen, RC	258	2373
5	Burnett, AL	213	3175
6	Musicki, B	202	2633
7	Cellek, S	167	2646
8	Angulo, J	165	2022
9	Goldstein, I	163	2161
10	Andersson, KE	163	2127

### Journal distribution

**Figure 7:**
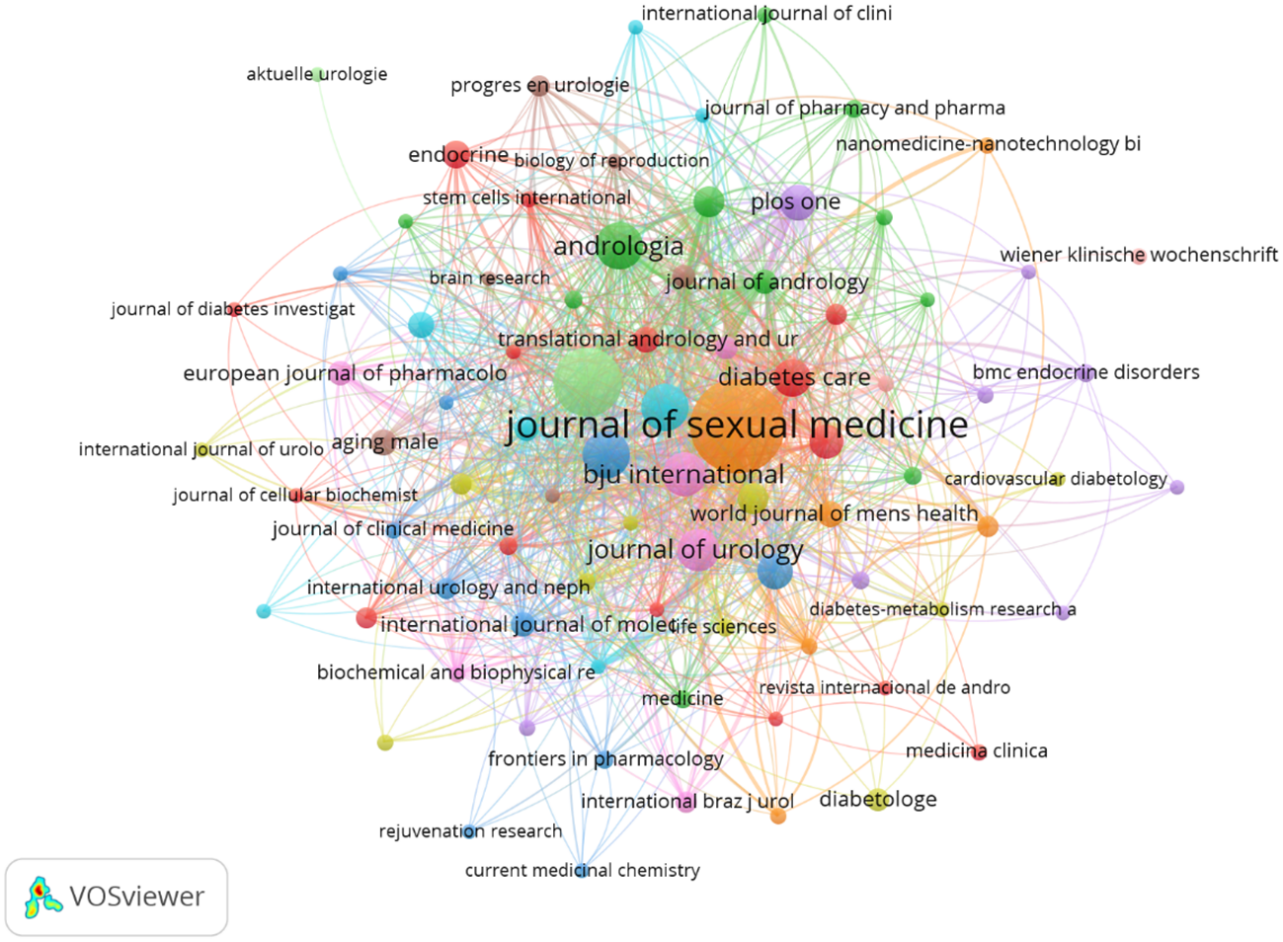
Analysis of cited journals.

The selected papers were published in a total of 359 journals. The 10 journals with the most publications were the Journal of Sexual Medicine (156), the International Journal of Impotence Research (77), Andrologia (36), the Asian Journal of Andrology (35), Andrology (35), BJU International (30), the Journal of Urology (29), Diabetes Care (21), Urology (20), and PLoS One (19) (**Table 7**; **Figure 7**).

**Table 7:** The 10 most productive journals.

Rank	Source	Documents	Citations	IF/JCR (2022)	Total link strength
1	Journal of Sexual Medicine	156	5048	3.937/Q3	1051
2	International Journal of Impotence Research	77	1893	2.408/Q3	549
3	Andrologia	36	356	2.532/Q3	178
4	Asian Journal of Andrology	35	624	3.054/Q1	323
5	Andrology	35	410	4.674/Q1	288
6	BJU International	30	1000	5.969/Q1	179
7	Journal of Urology	29	1277	7.600/Q1	274
8	Diabetes Care	21	5982	17.152/Q1	215
9	Urology	20	351	2.633/Q3	89
10	PLoS One	19	494	3.752/Q2	173

IF, impact factor; JCR, journal citation report.

A total of 4,634 journals were identified as being co-cited. The top five co-cited journals were the Journal of Sexual Medicine (3,656), the Journal of Urology (2,878), the International Journal of Impotence Research (2,058), Diabetes Care (1,676), and Urology (997) (**Table 8**).

**Table 8:** The 10 most co-cited journals.

Rank	Source	Citations	Total link strength
1	Journal of Sexual Medicine	3656	119103
2	Journal of Urology	2878	96586
3	International Journal of Impotence Research	2058	76294
4	Diabetes Care	1676	60565
5	Urology	997	37548
6	BJU International	970	39096
7	European Urology	956	34676
8	Diabetes	765	37672
9	Diabetologia	748	35252
10	New England Journal of Medicine	711	27729

### Analysis of highly cited and co-cited literature

**Figure 8:**
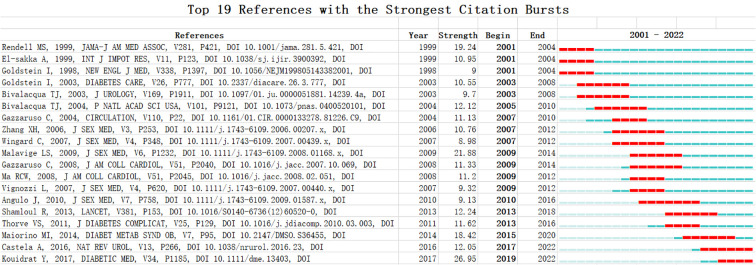
The 19 references with the strongest citation bursts.

A total of 1,168 references and 29,269 co-cited references were obtained. Some papers were cited over 300 times, including those by Tesfaye (2010), Boulton (2005), Vinik (2003a), Gallagher (2007), Jurenka (2008), Jones (2011), and Kapoor (2007b) (**Table 9**). In addition, a total of 19 references were obtained to highlight the analysis results. The three references with the highest intensities were Kouidrat Y, 2017 (doi: 10.1111/dme.13403), citation burst strength: 26.95; Malavige LS, 2009 (doi: 10.1111/j.1743-6109.2008.01168.x), citation burst strength: 21.88; and Rendell MS, 1999 (doi: 10.1001/jama.281.5.421), citation burst strength: 19.24 (**Figure 8**).

**Table 9:** The number of citations for the top 10 references.

Rank	Document	Citations	Links
1	Tesfaye (2010)	1389	7
2	Boulton (2005)	1207	7
3	Vinik (2003a)	1157	7
4	Gallagher (2007)	510	3
5	Jurenka (2008)	395	0
6	Jones (2011)	387	2
7	Kapoor (2007b)	342	5
8	Bivalacqua (2004)	266	21
9	Malavige (2009)	256	21
10	Gandaglia (2014)	250	3

### Keyword analysis

**Figure 9:**
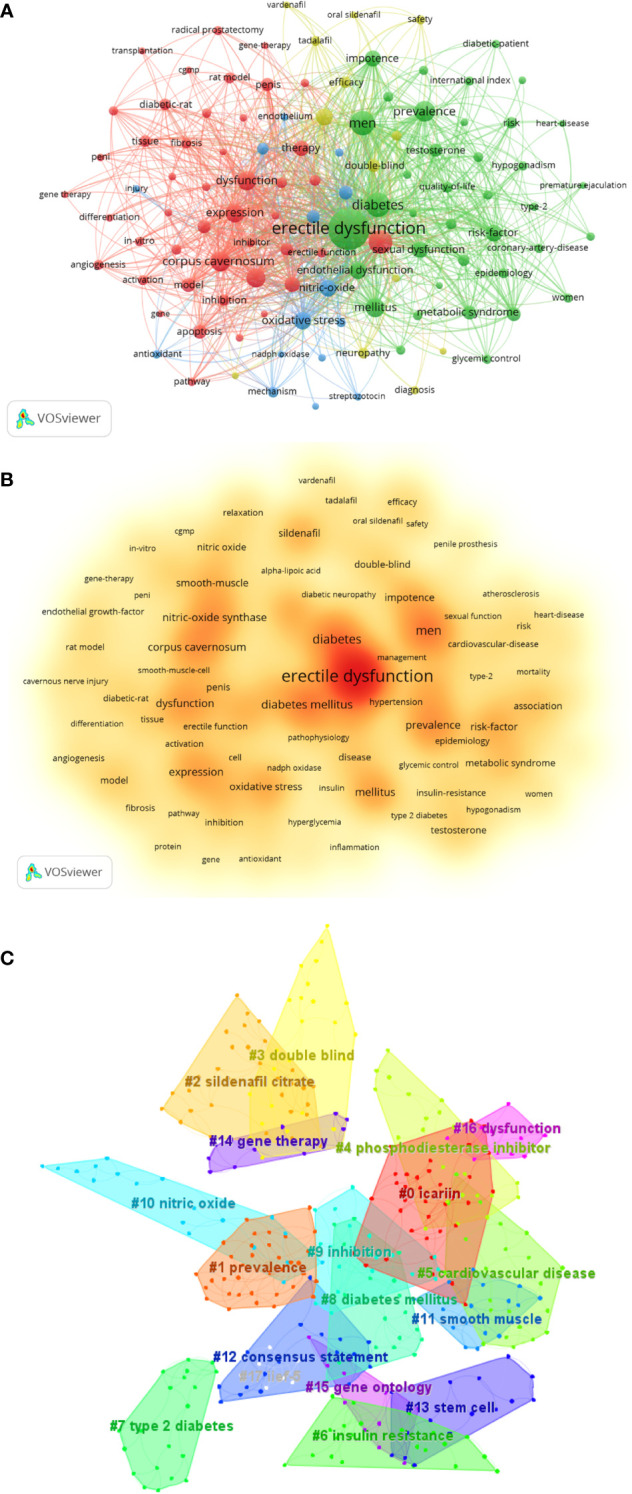
Keyword analysis. **(A)** Map of the keyword co-occurrence analysis obtained using VOSviewer. The size of the nodes represents the number of occurrences; the thickness of the curve represents the strength of collaboration; the different colors represent the different clusters. **(B)** Keyword density visualization analysis. The redder the node color, the higher the frequency of the keyword. **(C)** Keyword clustering map analysis using CiteSpace. A total of 16 categories of keywords were obtained; the different color blocks represent different keyword clusters.

Changing trends in research topics over time can be identified through keyword co-occurrence and salience analysis to better grasp the development of research hotspots. A total of 3,969 keywords were obtained, the top 10 of which were erectile dysfunction (796), men (256), diabetes (254), diabetes mellitus (239), prevalence (180), corpus cavernosum (171), dysfunction (155), mellitus (154), nitric oxide synthase (153), and expression (140) (**Table 10**; **Figure 9**).

**Table 10:** Top 10 keywords related to the field of assisted reproduction.

Rank	Keyword	Occurrences	Total link strength
1	Erectile dysfunction	796	4150
2	Men	256	1471
3	Diabetes	254	1503
4	Diabetes mellitus	239	1392
5	Prevalence	180	1004
6	Corpus cavernosum	171	1136
7	Dysfunction	155	930
8	Mellitus	154	961
9	Nitric oxide synthase	153	951
10	Expression	140	892

**Figure 10:**
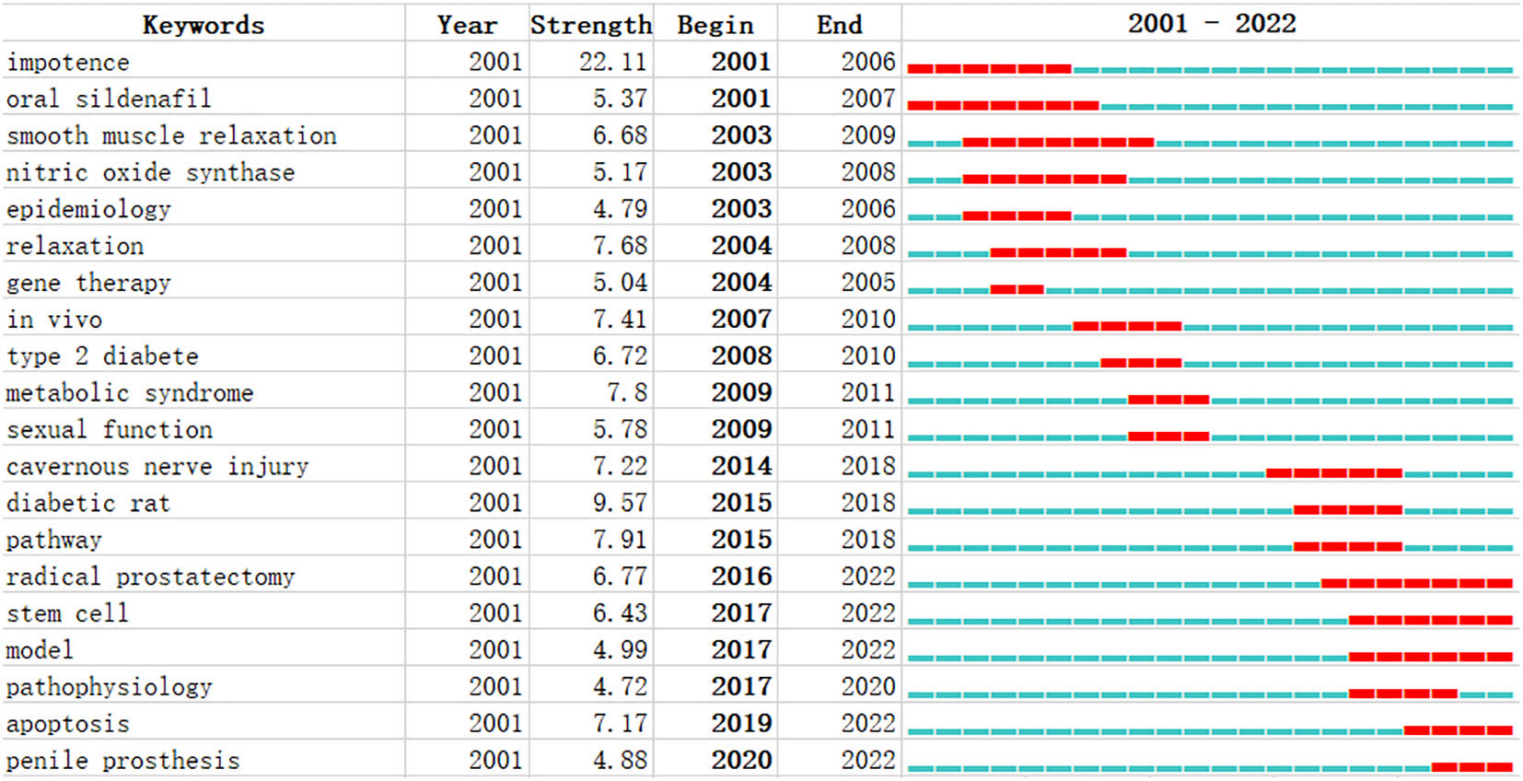
Keyword burst analysis using CiteSpace.

After clustering using CiteSpace software, a total of 18 categories of keywords were obtained. From 2001 to 2011, keyword-based research hotspots in the DMED field mainly included impotence, oral sildenafil, smooth muscle relaxation, nitric oxide synthase, epidemiology, relaxation, gene therapy, *in vivo*, type 2 diabetes, metabolic syndrome, and sexual function. From 2014 to 2022, meanwhile, the research hotspots (by keyword) included cavernous nerve injury, diabetic rat, pathway, radical prostatectomy, stem cell, apoptosis, model, pathophysiology, and penile prosthesis, among others (**Figure 10**).

## Discussion

Comprehensively and systematically summarizing the research topics, research trends, and global research status relating to DMED allows a rapid preliminary understanding of the research status of this condition. With the advent of big data, researchers need to fully understand the developments in their research field. Different from systematic review or meta-analysis, bibliometric analysis uses visualization software, such as VOSviewer and CiteSpace, to comprehensively analyze the existing literature and obtain an intuitive understanding of the research trends, as well as to predict research hotspots (16). This study is the first to summarize the research status relating to DMED in the past 20 years through bibliometric analysis.

### General information about the literature on diabetic erectile dysfunction

Over the past 20 years, the number of DMED-related articles published in journals showed a linear upward trend (*R* ^2 =^ 0.6709).

From the perspective of countries/regions and institutions, the number of publications originating from the United States as well as the number of associated citations far exceeded that of other countries. Although the number of articles published in China ranked second, the number of citations was low, ranking only fourth. This indicates that, although the number of papers from Chinese institutions is increasing yearly, there is still a lack of high-quality articles. This may be partly explained by a lack of cooperation with internationally renowned researchers as well as language barriers. Among the 10 institutions with the most published papers, only Huazhong University of Science & Technology, Peking University, and Nanjing University were from China—with the remainder being mainly from Europe and the USA—and these institutions had low levels of research cooperation. These observations highlight the need to strengthen communication and cooperation with global research teams, especially those from countries and institutions in the Asian region.

Regarding authors and references, Ryu, Ji-Kan and Suh, Jun-Kyu had the greatest production efficiency, while Bivalacqua, TJ had the most co-citations. Corona, G; Feldman, HA; Rosen, RC; and Burnett, AL also made important contributions to DMED research. The research undertaken by Ryu, Ji-Kan and Suh, Jun-Kyu is mostly mechanism-related. The former has mainly focused on identifying potential target genes associated with DMED in mouse cavernous pericytes (17). Meanwhile, Suh, Jun-Kyu found that pericellular-derived extracellular vesicles mimicking nanovesicles can promote neurovascular regeneration and erectile function recovery under diabetic conditions through a lipid chain enzyme 2 (LCn2)-dependent mechanism (18). Both authors reported that the preservation of damaged cavernous neurovasculature through the inhibition of the proNGF/p75NTR pathway may represent a novel therapeutic strategy for radical prostatectomy-induced erectile dysfunction (19).

The Journal of Sexual Medicine published the most DMED-related papers and the 10 most cited and co-cited journals were mainly in Q1 and Q2. This indicates that most papers published in this field were high-quality scientific research achievements.

### Hotspots and frontiers

Our analysis further demonstrated that the most influential authors and references were mostly associated with review articles and clinical guidelines from internationally renowned institutions and journals. Combining keyword co-occurrence, clustering, and salience analysis, we identified oral sildenafil, smooth muscle relaxation, acute oxide synthase, gene therapy, metabolic syndrome, cavernous nerve injury, stem cell, and penile prosthesis as the main research topics and hotspots in the field of DMED.

The main treatment for erectile dysfunction is drug therapy, including phosphodiesterase-5 (PDE5) inhibitors, androgen therapy, and vasoactive drugs (20–22). PDE5 inhibitors, such as sildenafil, tadalafil, and vardenafil, are the first-line oral drugs recommended by the World Health Organization (WHO) for the treatment of erectile dysfunction, including that associated with diabetes (23–25). These drugs increase the concentration of cyclic guanosine monophosphate (cGMP) in vascular smooth muscle cells by inhibiting PDE5 expression in the corpus cavernosa, reduce the concentration of intracellular calcium, cause smooth muscle relaxation, increase blood flow to the corpus cavernosa, and improve erection. Treating DMED with a PDE5 inhibitor can improve the International Index of Erectile Function-5 (IIEF-5) score and sexual success in a significant number of patients (26, 27).

Nevertheless, PDE5 inhibitors are ineffective in patients with severe erectile dysfunction (28) and are occasionally associated with side effects such as headaches, flushing, indigestion, nasal congestion, vision abnormalities, and diarrhea. The prerequisite for PDE5 inhibitor to function is that ED patients must have intact molecular and neurological pathways and some degree of sexual stimulation. Therefore, PDE5-Is has poor efficacy in patients lacking upstream nitric oxide (NO) pathway (29) in some disease states, such as diabetes with peripheral neuropathy (30) and prostate cancer (31). In addition to drugs, other alternative strategies are available, such as vacuum contractile devices and penile prosthesis implantation The three-piece inflatable penile prosthesis (IPP) is the most common implant used in penile surgery (32).The satisfaction rate of patients is 90% ~ 100%, which varies with different prosthesis devices (33, 34).However, IPP may only be offered to patients who fail due to the high cost of conservative treatment, its invasive nature, and myriad potential complications. Thus, novel therapeutic approaches are urgently required to overcome these disadvantages in DMED treatment.

Stem cell therapy has attracted increasing attention owing to its ability to promote functional recovery and tissue structural repair in patients with diabetes. Stem cell mainly include bone marrow mesenchymal stem cells (BMSCs) (35), adipose-derived stem cells (ADSCs) (36), neural derived stem cells (37), mesenchymal stem cells (MSCs) (38), umbilical cord derived stem cells (39) and urine-derived stem cells (USCs) (40).Mesenchymal stem cells (MSCs) are adult stem cells derived from mesoderm with a high potential for proliferation, self-renewal, and multidirectional differentiation (41). The culture of bone marrow-derived MSCs is relatively simple; accordingly, they are widely used in animal studies and clinical trials (42). Given their abundant autologous availability, MSCs are one of the most promising candidates for the treatment of DMED (43). Importantly, allogeneic MSCs can also be successfully transplanted owing to the low immunogenicity (44, 45). In animals, bone marrow-derived MSC transplantation can significantly improve the recovery of erectile function by inhibiting apoptosis (46). For DMED, MSC-based treatment has also attained encouraging therapeutic effects (47). The restoration of erectile function is mainly attributed to the increased endothelial cell and smooth muscle content in the corpus cavernosum (48, 49). Various strategies have recently emerged to improve the therapeutic effect of MSCs on DMED (50, 51).In a single-blind study, 6 out of 7 ED patients developed morning erections 3 months after treatment with umbilical cord derived stem cells (39).Chen S et al. found that adipose mesenchymal stem cells were more effective in treating diabetes-related erectile dysfunction than bone marrow mesenchymal stem cells (52).

Erectile dysfunction has been associated with the upregulation of Toll-like receptor 4 (TLR4) expression. It has been reported that TLR4 expression is significantly increased in the corpus cavernosum of diabetic rats compared with that of controls (53). The overexpression of inducible nitric oxide synthase (iNOS) may also be associated with penile microvascular dysfunction in diabetes mellitus, while endotoxemia has been linked with iNOS upregulation (54). Studies on conducting vessels have shown that iNOS overexpression in endotoxemia contributes to endothelial dysfunction by reducing the activity of endothelial nitric oxide synthase (eNOS) (55). iNOS inhibitors can alleviate DMED-related injury by moderating eNOS phosphorylation and chronic iNOS overexpression as well as improving microvascular fibrosis (56).

Trabecular smooth muscle relaxation during sexual stimulation and dilatation of penile resistance arteries in the cavernous body are essential for penile erection (57). Nitric oxide (NO) is a key factor in both processes. NO can be released from nerve endings or endothelial cells to stimulate cGMP production in penile smooth muscle cells, relaxing them and increasing blood flow to the corpus cavernosum (58, 59). Defects in the NO/cGMP pathway at any level will lead to inadequate penile smooth muscle relaxation, which affects erectile function (60).

*In vitro* low-intensity shockwave therapy (LI-SWT) is a potential treatment option for ED. The micro-damage of Li-SWT to the spongy tissue may stimulate neovascularization and up-regulate some factors related to tissue healing and remodeling (61).A prospective, randomized, sham controlled study reported a clinically significant improvement in erectile function in 40.5% of the treatment group based on the minimum clinically significant difference (MCID) criteria (62).Another randomized clinical trial evaluated changes in penile hemodynamics and IIEF-EF scores in patients with vascular ED. In the IIEF-EF score, 56.7% of the treatment group achieved MCID at 1 month and 75% achieved MCID at 12 months (63).

Through keyword co-occurrence and salience analysis, we also found that there is a regional imbalance in the development of DMED research and influential authors and institutions are mainly concentrated in Europe and Asia, which may be due to greater research interest. Additionally, despite the numerous studies undertaken on DMED, the underlying mechanism remains incompletely understood and represents a key future research direction.

### Limitations and prospects

To the best of our knowledge, this is the first bibliometric analysis of DMED-related studies undertaken in the last 20 years. However, our study still had some limitations. First, high-quality articles published in recent years may not have yet reached the ideal citation threshold, which may have introduced research bias. Second, there may be a delay in exploring the frontiers of research. Finally, we only included literature written in English from the WOS, which may have resulted in the omission of important documents in other languages.

### Conclusions

Overall, our bibliometric analysis provides comprehensive information on DMED-related publications. Our findings suggested that the field of DMED is thriving and has stimulated great interest in the research community worldwide. Although DMED is a relatively common disease, much remains unknown about both the underlying mechanisms and how to treat it. Likely, the terms oral sildenafil, smooth muscle relaxation, nitric oxide synthase, gene therapy, metabolic syndrome, cavernous nerve injury, stem cell, and penile prosthesis will be at the forefront of DMED-related research.

## Data availability statement

The original contributions presented in the study are included in the article/supplementary material, further inquiries can be directed to the corresponding authors.

## Author contributions

FM and HL (10^th^ author) designed the study. XL, HC, LW, MZ, HL (7^th^ author), GG and DL conducted the literature search. FM, XL and LW analyzed the data and wrote the paper. FM and JW approved the final manuscript. All authors contributed to the article and approved the submitted version.
